# Ubiquitination regulates allergic asthma by affecting immune cells and immune responses

**DOI:** 10.1016/j.bbrep.2025.102212

**Published:** 2025-08-19

**Authors:** Zheng Jin, Zhenhua Zhu, Xiaopeng Jing, Ji Zeng, Dongmei Yan

**Affiliations:** a Department of Clinical Laboratory, Wuhan Fourth Hospital, Wuhan City, Hubei Province, China; b Department of Joint Surgery, Wuhan Fourth Hospital, Wuhan City, Hubei Province, China; c Department of Immunology, College of Basic Medical Sciences, Jilin University, Changchun City, Jilin Province, China

**Keywords:** Allergic asthma, Ubiquitination, Immune cells, Immune responses

## Abstract

Allergic asthma, known for its airway hyperresponsiveness and remodeling, is a prevalent chronic respiratory disease. Recent investigations have emphasized the crucial role of ubiquitination, a post-translational modification, in the pathogenesis of allergic asthma. Ubiquitination involves the addition of ubiquitin molecules to substrates, caused their degradation or alteration in activity. Ubiquitination affects various aspects of immune cell function, such as activation of Th2 cells, B cells, and antigen-presenting cells, which are vital to allergic asthma.

In this review, we explore the role of ubiquitination in modulating immune responses during allergic asthma. We discuss the interplay between ubiquitin ligases, their substrates, and the impact on immune cell function, including Th2 differentiation and Th2 cytokines production. Our study also considers the potential therapeutic outcomes of targeting ubiquitination in asthma management. By understanding the complex interplay between ubiquitination, immune cells and immune responses, we can identify new molecules for treating allergic asthma, potentially leading to more effective therapies that modulate immune responses and ameliorate disease symptoms.

## Highlights

**Topic Overview**

• This review explores the role of ubiquitination in allergic asthma, focusing on its impact on immune cells and immune responses.

**Key Themes**

• We discuss the mechanisms by which ubiquitin ligases and deubiquitinating enzymes regulate Th2 cell differentiation, B cell function, and dendritic cell activity.

**Current Understanding**

• Recent studies have identified several key ubiquitin regulators, including USP38, USP7, MARCH1, and Cbl-b, that influence allergic inflammation and airway hyperresponsiveness (e.g., "USP38 stabilizes JunB to potentiate Th2 inflammation in asthma").

**Future Directions**

• Further research is needed to elucidate the specific molecular pathways involved and to develop targeted therapies that modulate the ubiquitin system for asthma management.

## 1 Introduction

Allergic asthma is a complex immunological disorder. It greatly impacts the millions of people globally. Allergic asthma stems from the imbalance of immune cells and immune responses, with the disturbed interplay between innate and adaptive immunity [1].

Ubiquitination, a critical post-translational modification, has emerged as a pivotal regulator in immunity [2]. This modification not only targets proteins for degradation in the 26S proteasome, but also mediates non-degradative signaling [3]. These signaling pathways are crucial for immune cell development, activation, and differentiation [2].

In allergic asthma, the balance of T helper 2 (Th2) cells drives allergic inflammation, which is regulated by ubiquitination [4]. Furthermore, ubiquitination affects antigen-presenting cells (APCs) that responsible for initiating and maintaining immune responses [5]. Ubiquitination is a pivotal regulator of immune cells and immune responses in allergic asthma.

Current researches focus on how ubiquitination-related proteins modulate allergic asthma, yet the cell-type-specific mechanisms underlying ubiquitination remain largely undefined. Exploring how ubiquitination affects these processes could lead to new therapies targeting the ubiquitin system for allergic asthma. This review will delve into the current knowledge of how ubiquitination affects immune cells and immune responses in allergic asthma, highlighting the potential targeted interventions to restore immune balance and alleviate disease pathology.

## 2 The role of ubiquitination in immunity

### 2.1 Ubiquitin and ubiquitination

Ubiquitination is a process where ubiquitin attaches to proteins via enzymes, often linked to degradation or modification [6]. In addition to substrate proteins, the molecules involved in ubiquitination include ubiquitin, E1 ubiquitin-activating enzyme, E2 ubiquitin-binding enzyme, and E3 ubiquitin-ligase. Comprising 76 amino acids and weighing about 8.5 kDa, ubiquitin is a small, highly conserved protein present in almost all eukaryotic life forms. The full length of ubiquitin contains seven lysine (Lys) sites (K6, K11, K27, K33, K48, and K63), one methionine site (Met1) at the N-terminal, and one glycine (Gly) site (G76) at the C-terminal. These sites allow ubiquitin molecules to connect, creating different ubiquitin chains. When ubiquitins are linked in the same manner, it forms homotypic ubiquitin chains; if not, it forms heterotypic ubiquitin chains [7]. Ubiquitin chains may be linear or develop into branched structures. During ubiquitination, ubiquitin is initially activated by E1 before being passed to E2. E3 binds with ubiquitin-loaded E2 and the substrate protein, forming iso-peptide bonds between ubiquitin's C-terminus and substrate lysine, modifying the target protein. The specific recognition of target protein is determined by E3 [8].

**Fig. 1:**
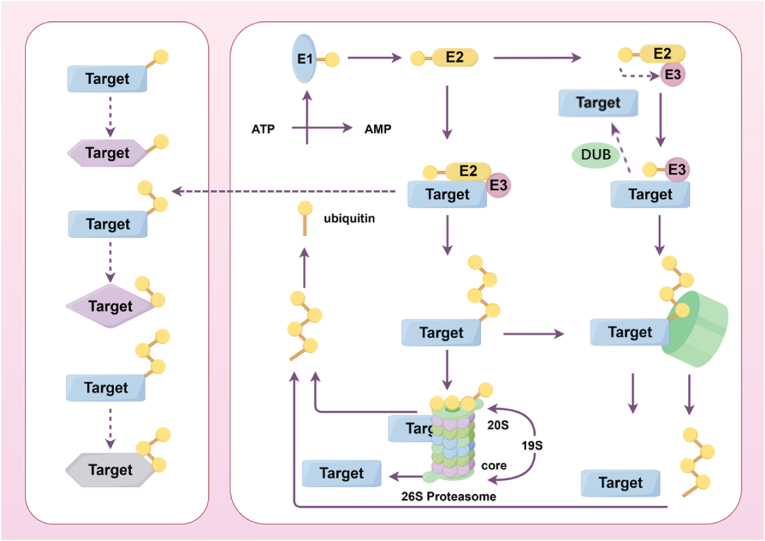
**Ubiquitination process.** Ubiquitination mainly includes ubiquitin activation, ubiquitin conjugation and ubiquitin ligation. The E3 enzyme recognizes the target protein and catalyzes the transfer of ubiquitin from the E2 enzyme to the lysine residues of the target protein, forming an iso-peptide bond. The E3 enzyme is key in the ubiquitination process because it determines the target protein for ubiquitination. Ubiquitination can be mono-ubiquitination or polyubiquitination. The formation of polyubiquitin chains usually leads to the recognition and degradation of the protein by the 26S proteasome. Ubiquitination is reversible, with the existence of deubiquitinating enzymes, which can remove ubiquitin molecules from proteins and regulate the level of ubiquitination. By Figdraw.

At present, 2 E1s (UBA1 and UBA6), about 35 E2s and more than 600 E3s have been identified [9,10]. According to the binding site of ubiquitin to the substrate, there are currently 9 ways of ubiquitination. Different modes of ubiquitination regulate different activities. Among them, ubiquitin associated with proteasome degradation is converted to K48. K48 chains represent the majority of ubiquitin connections, constituting around 50 % of the total [11]. RNA and DNA damage may be linked to the K6 ubiquitin chain [12], as well as mitochondrial autophagy [13]. The Lys48/Lys11 branch chain formed by the combination of K11 and K48 can enhance the degradation of the proteasome, and the K11 ubiquitin chain can also regulates the cell cycle [14]. The recruitment of proteins in the DNA damage response (DDR) can be facilitated by K27-linked ubiquitin chains serving as scaffolds [15]. K29 ubiquitin chain can regulate lysosome degradation [16]. K33-linked chain participates in biological processes, for example, T cell surface receptors are marked by K33-linked ubiquitination, providing a signal for receptor-mediated signal transduction via non-endocytic pathways [17]. DNA repair and endocytosis may involve modifications of ubiquitin chains and monoubiquitin linked to K63 [18]. The linear ubiquitin chain linked to Met1 can serve as a crucial positive regulator of nuclear factor kappa-B (NF-κB) signaling and is significant in tumor, inflammation, and immune responses [19]. In summary, ubiquitination regulates biological processes through various forms (Fig. 1).

SUMOylation is a post-translational modification of proteins and is also a type of ubiquitination-like modification. It involves the covalent attachment of small ubiquitin-like modifiers (SUMO) to lysine residues on substrates [20]. In mammals, three main SUMO subtypes have been identified, including SUMO1, SUMO2, and SUMO3. Similar to ubiquitination, SUMOylation is catalyzed by a series of enzymes, including E1 SUMO-activating enzymes (SAE1/SAE2), a single E2 conjugating enzyme (UBC9), and a limited number of E3 SUMO ligases. SUMOylation mediates the targeting and functional regulation of target molecules [21]. Currently, the role of SUMOylation in cell biology is very extensive and is one of the hot topics of current research. An imbalance between SUMOylation and de-SUMOylation is associated with the occurrence and development of various diseases, including cancer, neurodegenerative diseases, heart disease, and innate immune diseases [22] [[22], [23]].

Deubiquitination plays a vital role in cells by counteracting the ubiquitin-mediated post-translational modifications of proteins [24]. Deubiquitinating enzymes (DUBs) remove ubiquitin from proteins, regulating the ubiquitin-proteasome system (UPS) and impacting protein lifespan and integrity. These enzymes can remove single ubiquitin units and polyubiquitin chains from proteins, sever peptide or isopeptide bonds, and release free monoubiquitin [25]. DUBs are linked to various human diseases like cancers and neurodegenerative disorders, making them key candidates for therapeutic development [26] (Fig. 1).

### 2.2 Ubiquitination in immunity

Ubiquitination regulates protein stability and function, impacting innate and adaptive immunity, as well as tumor and inflammation responses. Modulating immune checkpoint pathways by targeting the ubiquitination/deubiquitination process could be a potential therapeutic target for cancers, infections, and autoimmune diseases [27]. By inhibiting T cell activation and proliferation, programmed cell death 1 (PD-1)/programmed cell death-ligand 1 (PD-L1) reduced the chances of ongoing autoimmune inflammation. PD-1 and PD-L1 on tumor cells inhibit T cells, preventing recognition and destruction of tumors, allowing them to evade immune surveillance [28]. There were some E3s can regulate PD-1/PD-L1 in tumor immunotherapy. For example, continuous photomorphogenic 1 (COP1) facilitated PD-L1 expression by promoting histone H3 acetylation, thus driving tumor progression [29].

Ubiquitination regulates cell signaling by forming multiple types of polyubiquitin chains, such as K6, K11, K27, K29, K33, K48, and K63. These different ubiquitin chain types are structurally and functionally different, and they act as signals for different processes within the cell. As an E3 ubiquitin ligase, tripartite motif containing (TRIM) 25 promoted NF-κB signaling via tumor necrosis factor (TNF)-α by interacting with TNF receptor associated factor 2 (TRAF2), enhancing its K63-linked polyubiquitination, and connecting it to transforming growth factor-β (TGF-β)-activated kinase 1 (TAK1) or inhibitor of kappa B kinase β (IKKβ), thus boosting NF-κB activation [30].

Ubiquitination plays a key role in the development and function of immune cells, particularly in T cell activation and inflammatory responses. Dendritic cells (DCs), as significant antigen-presenting cells, rely on MHC Class II (MHCII) molecules on their surface to activate CD4^+^ T cells. Membrane associated RING-CH (MARCH) 1 is an E3 ubiquitin ligase. MARCH1 regulated the intracellular transport and degradation of MHCII and other molecules through ubiquitination, affecting the antigen presentation process [31]. At the same time, unubiquitinated MHCII led to decreased expression of MHCI and inhibited the activation of CD8^+^ T cells [32].

## 3 Immunity in allergic asthma

Asthma prevalence is rising, with 262.41 million cases globally. Only 28.8 % of 2032 sampled asthma patients were diagnosed, indicating many undiagnosed cases [33]. According to the most recent meta-analysis, the overall prevalence of allergic asthma in Mainland China was 2.20 %. The prevalence of asthma has risen over the last thirty years, from 0.69 % in 1984 to 5.30 % in 2021. Projections showed that by 2050, the prevalence of the disease in the Chinese population could reach 9.76 % [34]. It is necessary to understand the mechanisms and mitigation strategies for allergic asthma.**(see** Table 1**)**

**Table 1:** The role of E3 ubiquitin ligase in allergic asthma.

E3 ubiquitin ligase	Targeted cell	Targeted protein	Effect	Mechanism	Reference
CBX4	Epithelial cells	TFII-I	Exacerbated	Enhanced the SUMOylation of TFII-I, thereby promoted the transcription of MEX-3B and translation of lfTSLP, which in turn aggravated inflammation	[48]
CBX4	Epithelial cells	β-catenin	Exacerbated	Disrupted epithelial cell function	[49]
PIAS1	Goblet cell	ROCK2	Exacerbated	Promoted the SUMOylation of ROCK2 at the K1007 site, enhanced its binding and activation with RhoA, and promoted goblet cell metaplasia	[50]
PARK2	Bronchial epithelial cells	NLRP3	Relieved	Mitigated HDM-induced NLRP3 inflammasome activation, inflammatory factor release, pyroptosis, and airway epithelial cell barrier damage by ubiquitinating NLRP3	[51]
MID1	Bronchial epithelial cells	PP2Ac	Exacerbated	Conjugated and ubiquitinated PP2Ac, thereby affected its stability and activity, promoted the phosphorylation of IκBα as well as p38 MAPK and JNK	[52]
Fbw7	Th2	GATA3	Relieved	Induced GATA3 degradation through proteasome pathway, thus inhibiting Th2 cell differentiation	[60]
Cpl-b	Th2/9	STAT6	Relieved	Bond to and ubiquitinated STAT6 for degradation at sites K108 and K398, thereby inhibiting the Th2 response, and it suppressed the Th9 response through both STAT6-dependent and independent ways	[63]
Cul5	CD4^+^ T	*p*-JAK1	Relieved	Inhibited the ubiquitination and degradation of *p*-Jak1, led to decreased *p*-Jak1 and p-STAT6, which in turn inhibiting the differentiation of Th2 or Th9	[68]
Grail	Th2	STAT6	Relieved	Inhibited the differentiation of Th2 by interacting with STAT6 and promoting its ubiquitination and degradation	[70]
A20	CD4^+^ T	GATA3	Relieved	Inhibited the differentiation of Th2	[71]
AMFR	AM	CIS	Exacerbated	Bond to CIS and induced Lys48 chain polyubiquitination of CIS, blocked the inhibition of CIS on STAT5 phosphorylation and the activation of its downstream pathway, thereby promoting the production of GM-CSF	[75]
Cul5	AM	OGT	Exacerbated	Interacted with OGT, inducing Lys48-linked polyubiquitination of OGT, and promoted neutrophil inflammation	[76]
SOCS1	AM	IRS-2	Relieved	Promoted the ubiquitination of IRS-2, inhibited its tyrosine phosphorylation, negatively regulated the differentiation of M2 macrophages	[77]
NEDD4.2	AM	IL-4R	Relieved	Interacted with IL-4 receptor, promoted its ubiquitination and degradation, thereby negatively regulating IL-4-induced IRS-2 signaling	[78]
MARCH1	DC	MHC II, CD86	Exacerbated	Mediated the ubiquitination and degradation of MHC II and CD86, affected the antigen-presenting capacity of DCs to T cells, and promoted the differentiation of Th2	[79,80]
MARCH2/3	Eosinophils	IL-5Rα	Relieved	Bond to IL-5Rα and mediated its K27 chain polyubiquitination at K379 and K383 sites, followed by lysosomal degradation, inhibited the infiltration of inflammatory cells, peri-bronchial mucus secretion, and the production of Th2 cytokines	[84]
Nedd4-2	Mast cells	*p*-Syk	Relieved	Mediated the ubiquitination of *p*-Syk to limit the intensity and duration of IgE-FcεRI-induced signaling	[88]
TRIM27	Mast cells	PI3KC2β	Relieved	Ubiquitinated and inhibited PI3KC2β activity to negatively regulate KCa3.1 channel activity and downstream signaling of FcεRI, thereby inhibiting mast cell activation and allergic responses	[89]

**Fig. 2:**
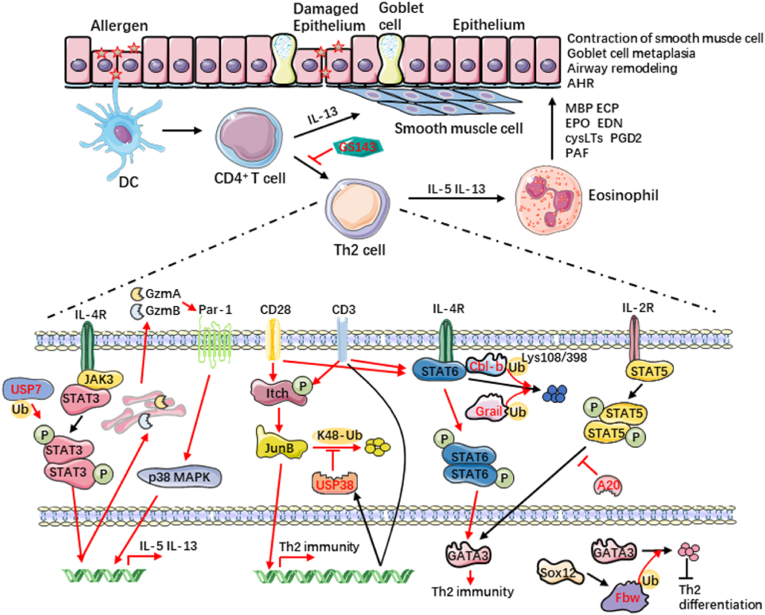
**Ubiquitination participates in Th2 immune response**. Allergic asthma is closely related to Th2-type immune responses. USP7 regulated the phosphorylation of STAT3, significantly influencing the secretion of IL-5 and IL-13. Deubiquitinating enzyme USP38 directly binds to JunB and removes TCR-induced polyubiquitination of JunB, maintaining the stability of JunB and promoting Th2 differentiation. Grail and Cbl-b interacted with STAT6, leading to its ubiquitination and degradation, thereby controlling the development of Th2. A20 inhibited GATA3 through IL-2-dependent mechanism, thereby alleviating Th2 immunity. Fbw7 induced GATA3 degradation through proteasome pathway, thus inhibiting Th2 cell differentiation. GS143 inhibited NF-κB activation and the expression of Th2 cytokines. MBP: myelin basic protein; ECP: eosinophil cationic protein; EPO: erythropoietin; EDN: endothelin; cysLTS: cysteinyl leukotrienes; PAF: platelet-activating factor.

Environmental allergens like house dust mite (HDM), fungi, pet dander, and pollen are often associated with the sensitization that leads to allergic asthma [35]. After these allergens are inhaled, they are captured by the airway epithelium and submucosal DCs [36]. Airway epithelial cells form a physical barrier through tight connections that prevent allergens and pathogens in the environment from penetrating. Airway epithelial cells release 'alarmin' cytokines like thymic stromal lymphopoietin (TSLP), interleukin (IL)-25, and IL-33 upon encountering allergens or irritants. These cytokines activate downstream immune cells, including Th2 cells and group 2 innate lymphoid cells (ILC2), triggering inflammation[[37], [38]] [[37], [38]]. And, in asthma, airway epithelial cells may oversecrete mucus, leading to airway blockage [39]. The allergen crosses the damaged epithelial barrier to reach DCs. DCs capture it, form MHC II-antigen complexes, and present them to CD4^+^ T cells via MHC II. Th2 cells and follicular helper T (Tfh) cells differentiate and secrete type 2 cytokines when co-stimulatory molecules like cluster of differentiation (CD)80, CD86, OX40 (CD134), and their ligands, along with cytokines such as IL-4, are present [40]. In lung tissue, type 2 cytokines promote the maturation and gathering of immune cells like eosinophils, ILC2s, DCs, macrophages, and NKTs. These cells drive inflammation, goblet cell metaplasia, and airway hyperreactivity (AHR) [41]. Upon exposure to the allergen, B cells produce IgE, which binds to the high-affinity Fc epsilon receptor I (FcεRI) on basophils and mast cells. When the allergen is encountered again, the cross-linking of allergen-specific IgE molecules with IgE leads to the degranulation of mast cells and basophils, releasing mediators such as histamine, prostaglandins, leukotrienes, cytokines, and chemokines [42,43]. These mediators are capable of contracting airway smooth muscle cells, leading to edema and mucus secretion. Chemokines attract a variety of inflammatory cells, including eosinophils, macrophages, neutrophils, and T lymphocytes. The products released by activated white blood cells set the stage for AHR and airway remodeling, resulting in epithelial damage, bronchoconstriction, and extracellular matrix deposition [44]. Over time, there may be lasting changes in airway structure, airway obstruction, and insufficient lung ventilation. Thus, various immune cells and cytokines are deeply involved in the occurrence and development of allergic asthma (Fig. 2).

## 4 Ubiquitination regulates allergic asthma through immune cells and immune responses

### 4.1 Epithelial cells

Airway epithelial cells serve as the primary defense mechanism for the respiratory tract. After exposure to allergen stimulation, airway epithelial cells participate in allergen sensitization and inflammatory stimulation process, which is also the first link to start bronchial asthma, and play an important role in the natural immune function of the lung [45]. Ubiquitination of proteins in epithelial cells can affect allergic asthma.

Sirtuin 3 (SIRT3) functioned as a mitochondrial deacetylase that significantly influenced mitochondrial metabolism, involving the tricarboxylic acid cycle, fatty acid oxidation, ect [46]. In HDM-induced allergic asthma, SIRT3 is a SUMOylation target. Increased SUMOylation of SIRT3 leads to its ubiquitination and proteasomal degradation, reducing its deacetylase activity and antioxidant function, and impairing airway epithelial barrier integrity and anti-inflammatory effects. SUMO specific protease 1 (SENP1) inhibited the SUMOylation of SIRT3, and β-nicotinamide mononucleotide (NMN) treatment promoted the SUMOylation of SIRT3 by activating SENP1, thereby stabilizing SIRT3 protein, alleviating epithelial cell damage [47].

Other studies also demonstrated that SUMOylation in epithelial cells regulated allergic asthma. Chromobox 4 (CBX4), as a SUMOylation E3 ligase, enhanced the binding of general transcription factor II-I (TFII-I) to muscle excess (MEX)-3B (MEX-3B) promoter by enhancing the SUMOylation of TFII-I, and enhancing the TFII-I -mediated RNA-binding protein MEX-3B transcription. MEX-3B promoted the translation of long-form TSLP (lfTSLP) by binding to its mRNA. In allergic asthma, lfTSLP promoted inflammation. Therefore, CBX4 might be a new target for lfTSLP-mediated asthma therapy [48]. There was a study that complement another mechanism of CBX. In HDM induced allergic asthma, CBX4 mediated the SUMOylation of β-catenin and deteriorated epithelial cell function [49].

One of the characteristics of allergic asthma is goblet cell metaplasia and subsequent hypersecretion of mucus. SUMOylation was significantly upregulated in bronchial epithelium in patients with allergic asthma and in mouse models. Protein inhibitor of activated STAT (PIAS) 1, an E3 ligase facilitated the SUMOylation of Rho associated coiled-coil containing protein kinase 2 (ROCK2) at the K1007 site, promoting allergen-induced airway inflammation, calytosis and AHR by promoting its binding and activation to Ras homolog gene family member A (RhoA) [50].

Similarly, Parkin (PARK2), an extremely versatile E3 ubiquitin ligase, was originally thought to be a neuroprotective gene that strongly associated with Parkinson's disease (PD). In HDM-induced airway epithelial cells, PARK2 was identified as a regulator that suppresses nucleotide-binding oligomerization domain, leucine-rich repeat and pyrin domain-containing 3 (NLRP3) inflammasome activation, inflammatory factor release, pyrodeath, and barrier damage via ubiquitination [51].

In the bronchial epithelium of mice, the E3 ubiquitin ligase midline 1 (MID1) increased following exposure to HDM or rhinovirus infection. MID1 could bind and ubiquitinate protein phosphatase 2Ac (PP2Ac), thereby affecting its stability and activity [52]. Activation of PP2A reduced phosphorylation of inhibitor of NF-κB (IκB) α, decreased NF-κB activity, and decreased phosphorylation of p38 mitogen-activated protein kinase (MAPK) and c-Jun N-terminal kinase (JNK), which were key inflammatory signaling pathways in asthma and other inflammatory diseases [53]. Thus, MID1 promoted allergic asthma by reducing PP2A activation [52].

### 4.2 T cells (Th1/Th2/Th9/Tregs)

A key factor in allergic asthma is the Th1/Th2 cells imbalance. Th2 cells in allergic asthma produce IL-4, IL-5, and IL-13, driving B cell differentiation, antibody production, eosinophil increase, and airway inflammation [54]. IL-5 is important in eosinophilic differentiation and survival, and it serves a pivotal function in eosinophilic inflammation in asthma [55]. IL-4 promotes B cell differentiation into IgE-producing plasma cells, which activate mast cells and basophils via allergen binding, releasing inflammatory mediators [56]. IL-13 acts directly on airway smooth muscle cells to increase airway sensitivity to various stimuli, leading to AHR [56]. Th1 cells secrete interferon (IFN)-γ to suppress Th2 cell differentiation and function, maintaining immune balance. Th1 cells may not have as strong an anti-inflammatory role in asthma as Th2 cells, but they help modulate the immune response and prevent excessive Th2 reactions [57] (Fig. 2).

During the initial phases of Th2 cell differentiation, there was a population of Th2 cells capable of producing serine proteases granzyme (Gzm) A and B. The Gzms had the capability to lyse protease-activated receptor (Par)-1 and promoted the phosphorylation of p38 MAPK. This mechanism boosted the generation of IL-5 and IL-13 in Th2 cells from both mice and humans. Ubiquitin-specific protease 7 (USP7) controlled the phosphorylation of STAT3 induced by IL-4. This process was essential for the production of granzymes during Th2 cell differentiation. The USP7-STAT3-Granzyme-PAR-1 axis significantly influenced Th2 cell differentiation by regulating the production and activity of GzmA and GzmB, as well as the phosphorylation of STAT3 by USP7, particularly in the production of IL-5 and IL-13, which played a key role in allergic diseases [58] (Fig. 2).

The main transcription factor for Th2 cells, GATA binding protein 3 (GATA3), underwent regulation via several modifications, including ubiquitination and deubiquitination [59]. SRY-related high-mobility-group (HMG)-box (SOX) 12 mRNA was significantly increased during Th2 cell differentiation. High expression of SOX12 decreased GATA3 in CD4^+^ T cells, but did not affect mRNA expression, suggesting that post-translational modification of GATA might occur. Further studies showed that SOX12 promoted GATA3 ubiquitination mediated by E3 ubiquitin ligase F-Box and WD repeat domain containing (Fbw) 7 and induced GATA3 degradation through proteasome pathway, thus inhibiting Th2 cell differentiation and development of allergic asthma [60] (Fig. 2).

Casitas B lymphoma-b (Cbl-b) functioned as an E3 ubiquitin ligase that catalyzes the formation of K33-linked ubiquitin chains. In T cells, Cbl-b attaches K33-linked ubiquitin to Zap70, dampening TCR signaling and preventing excessive immunity [61]. Effector T cells lacking Cbl-b were not inhibited by regulatory T cells. Cbl-b deficiency could alter the severity of allergic airway disease by causing an imbalance in T cell response, but the precise mechanism required further study [62]. Another study looked in detail at the role of Cbl-b in allergic asthma. In *vitro*, the loss of Cbl-b enhanced the differentiation of Th2 and Th9 cells, causing severe airway inflammation and more robust Th2 and Th9 responses in mouse asthma models. Under IL-4 stimulation, Cbl-b specifically interacted with STAT6, leading to the ubiquitination and degradation of STAT6 at the K108 and K398 sites. This process was enhanced by T cell receptor (TCR)/CD28 co-stimulation. Cbl-b regulated STAT6 through the ubiquitin-proteasome pathway, thereby inhibiting Th2 response, but inhibited Th9 response through STAT6-dependent and independent mechanisms. These findings revealed new understandings of Cbl-b's role in T cell differentiation and allergic airway inflammation, possibly contributing to the formulation of innovative therapeutic strategies [63] (Fig. 2).

USP38 is a DUB that specifically cleaves K33-linked ubiquitin chains; by removing K33 ubiquitin from TBK1, it promotes type I interferon pathway activation [64]. USP38 was a novel histone DUB, and its role in allergic asthma has been rarely studied [65]. Studies had shown that JunB played key role in Th2 cell differentiation, mediating allergic reactions and inflammation by promoting the expression of Th2-related cytokines such as IL-4 [66]. USP38 bond directly to JunB, removed polyubiquitination of JunB's Lys-48 link, thereby preventing TCR-induced JunB degradation. A significant reduction in Th2 cell numbers was observed due to USP38 defection, without impacting Th1, Th17, Tregs, and Th2 cytokines like IL-4, IL-5, and IL-13 were significantly reduced. USP38 promoted asthma pathogenesis by stabilizing JunB protein and was the first identified DUB specific to the Th2 immune response. USP38 might be a potential target for Th2 immune response and related asthma therapy [67] (Fig. 2).

Cullin 5 (Cul5) is an E3 ubiquitin ligase that regulates CD4^+^ T cell differentiation via IL-4 receptor signaling. Mice lacking Cul5 in CD4^+^ T cells showed Th2/Th9 inflammation and allergic asthma features. Cul5 deletion reduced JAK1 ubiquitination and degradation, increasing *p*-JAK1 and *p*-STAT6 levels. This made T cells hypersensitive to IL-4, promoting Th2/Th9 development and worsening asthma inflammation and pathology [68].

Grail is a type I transmembrane protein as well as an E3 ubiquitin ligase [69]. Increased Grail was expressed in Th2 cells, regulating their development by interacting with STAT6 and promoting its ubiquitination and degradation. Grail-deficient T cells showed increased Th2 effector cytokine expression both in *vitro* and in *vivo*. Mice lacking Grail were more susceptible to allergic asthma [70] (Fig. 2).

A20, or tumor necrosis factor alpha-induced protein 3 (Tnfaip3), is a ubiquitin editing enzyme that blocks NF-κB signaling activated by inflammatory stimuli. In A20-deficient CD4^+^ T cells, GATA3 expression was significantly increased, promoting Th2 cell differentiation without affecting Th1 or Th17 cells. A20 inhibited Th2 cell differentiation through its zinc finger domain's ubiquitin ligase activity [71] (Fig. 2).

Encoded by the Cyld gene, the Cylindromatosis (CYLD) protein functioned as a deubiquitinating enzyme that diminished the NF-κB signaling pathway's activity by removing active ubiquitin residues. T cells with a Cyld mutation produced excess IL-9 when differentiating into Th9 cells in vitro, worsening allergy symptoms [90].

### 4.3 B cells

In allergic asthma, IgE produced by B cells can induce mast cell degranulation, leading to bronchoconstriction [42] [43]. B cells switch the heavy chain of antibodies from IgM or IgD to IgE through a process called class switch recombination (CSR). IL-4 and IL-13 are key cytokines that induce IgE class switching. They promote the phosphorylation of STAT6 via their receptors, activating the STAT6 signaling pathway to enhance IgE production [[72], [73]] [[72], [73]]. PPARγ, an E3 ubiquitin ligase, can interacted and degraded p-STAT6, inhibiting IgE switching and synthesis. Prostaglandin E2 (PGE2)- E-prostanoid 4 (EP4) signaling up-regulated PPARγ expression by Akt activation, high expression of PPARγ degradated of *p*-STAT6 through ubiquitination. The downregulated *p*-STAT6 inhibits IgE class switching, thereby alleviating the strong contraction of the trachea in allergic asthma [74].

### 4.4 Macrophages

AMFR (autocrine motility factor receptor) was upregulated in alveolar macrophages (AMs), which played a key role in allergic asthma. Under TSLP stimulation, AMFR was directly bond to CIS (a cytokine induced protein containing SH2 domain) and induced its Lys48 poly-ubiquitination, blocking CIS inhibition of STAT5 and promoting GM-CSF production. This caused accumulated eosinophils and enhanced Th2 cell-mediated immune responses, exacerbated the inflammatory responses in asthma [75].

Cul5 also influenced allergic asthma by modulating the antiviral immune response of AMs. When interacted with O-GlcNAc transferase (OGT), Cul5 promoted Lys48-linked polyubiquitination of OGT, blocked OGT's role in activating mitochondrial antiviral signaling protein O-GlcNAcylation and RIG-I signaling, reduced type I IFN expression, and promoted neutrophil-induced inflammations in asthma exacerbation [76].

IL-4 binding to macrophage receptors via insulin receptor substrate 2 (IRS-2) signaling induces M2 macrophage differentiation, associated with severe asthma. Inhibitory cytokine signaling protein 1 (SOCS1) was highly induced in human monocytes under IL-4 stimulation. As an E3 ubiquitin ligase, SOCS1 facilitated the ubiquitination of IRS-2, hindered its tyrosine phosphorylation, negatively impacted the differentiation of M2 macrophages [77]. IRS-2 was also a target for other proteins that regulate allergic asthma. IL-4 stimulation led to an inverse correlation between tyrosine and serine phosphorylation of IRS-2. Increasing serine phosphorylation of IRS-2 and decreasing tyrosine phosphorylation occur when serine phosphatase activity was inhibited, thereby decreasing M2 gene expression. P70S6K was an effector molecule downstream of mammalian target protein complex of rapamycin 1 (mTORC1) that negatively regulated IRS-2 tyrosine phosphorylation induced by IL-4 through serine phosphorylation of IRS-2. Another regulatory protein downstream of mTORC1, GRB10, could suppress IL-4-induced IRS-2 signaling by enhancing the ubiquitination and degradation of these proteins via its interaction with the α and γ chains of IRS-2, NEDD4.2 (E3 ubiquitin ligase), and IL-4 receptors. Reducing GRB10 led to increased tyrosine phosphorylation of IRS-2 and elevated M2 macrophage, indicating that GRB10 negatively regulated the IL-4 signaling pathway. P70S6K and GRB10 inhibited the polarization of M2-type macrophages through different mechanisms, thereby alleviating allergic asthma [78].

### 4.5 DCs

MARCH1, an E3 ubiquitin ligase, downregulates MHCII and CD86 expression via ubiquitination. This controls their levels on DC surfaces, affecting antigen presentation to T cells and TCR signaling. MARCH1 deficiency increases MHCII and CD86 on DC surfaces, causing persistent TCR signaling in T cells. This leads to sustained CD69 expression, reduced GATA3, and increased T-bet, inhibiting Th2 and promoting Th1 differentiation[79], [80][79], [80] [[79], [80]]. Genome-wide association studies (GWAS) and expression quantity trait loci (eQTL) analysis showed that genetic variation in MARCH1 was associated with human asthma risk [80]. Another study made a similar observation. Through the ubiquitination of MHCII and CD86, MARCH1 governed their expression on the surface of APCs, preventing ongoing antigenic signaling by effector CD4^+^ T cells and avoiding T cell depletion [81]. It has been noted that MARCH1 might increase allergic responses by boosting the expression of the complement receptor CD88 (C5aR) on inflammatory cells [82]. Targeting MARCH1 might offer a novel approach for the prevention or treatment of diseases that stimulate type 2 immunity.

### 4.6 Eosinophils

IL-5 is crucial in asthma as it facilitates the maturation, activation, movement, and survival of eosinophils. Blocking IL-5 signaling is a treatment approach for asthma [83]. Researchers showed that MARCH2 and MARCH3 were significant negative regulators of IL-5 signaling pathways. They interacted with IL-5Rα and promoted polyubiquitination at the K27 linkage on K379 and K383, followed by lysosomal degradation. Lack of MARCH2 and MARCH3 worsened OVA-induced airway inflammation, causing more inflammatory cells, increased mucus secretion, and higher Th2 cytokine production [84].

Ubiquitination of the IL-5R's βc chain impacts endocytosis, degradation, and signaling. JAK kinases bind to βc at Lys457, Lys461, and Lys467, promoting βc's subcellular localization, IL-5-induced endocytosis, turnover, and signaling, thereby influencing eosinophil function in allergic asthma [85].

### 4.7 Mast cells

In IgE-dependent allergic conditions, mast cells had a significant role. IgE is produced by B cells and, by binding to FcεRI, triggers the activation of mast cells, leading to the release of inflammatory mediators such as histamine and leukotrienes, which cause airway hyperresponsiveness and inflammation [86]. Upon allergen stimulation, IgE cross-links with the allergen, activating downstream signaling pathways of FcεRI expressed on mast cells, such as the activation of Syk kinase. Syk promotes the phosphorylation of key MAPKs, activates Erk1/2, and mobilizes calcium, leading to mast cell degranulation and the release of histamine [87]. Nedd4-2 (an E3 ubiquitin ligase) and its adaptor Nedd4 family interacting protein 1 (Ndfip1) were negative regulators of IgE-FcεRI signaling. By ubiquitinating degrading phosphorylated Syk (*p*-Syk), Nedd4-2 restricted the intensity and length of IgE-FcεRI-induced signaling. Mast cells lacking Nedd4-2 or Ndfip1 exhibited increased release of histamine and pro-inflammatory cytokines in response to IgE, enhancing allergic reactions [88].

Phosphatidylinositol-3-kinase C2β (PI3KC2β) was required for FcεRI-activated KCa3.1 channels, Ca^2+^ influx, cytokine production, and bone marine-derived mast cell (BMMC) degranulation. TRIM27 negatively regulated KCa3.1 channel activity and downstream signaling of FcεRI through ubiquitination and inhibition of PI3KC2β activity. TRIM27 inhibited mast cell activation and allergic response [89].

## 5 Drugs affect allergic asthma by regulating ubiquitination

GS143 is a small molecule inhibitor that specifically blocks IκB ubiquitination, preventing NF-κB activation and target gene transcription [91]. Intranasal GS143 inhibited ovalbumin (OVA)-induced NF-κB activation, reduced airway eosinophils and lymphocytes, lowered Th2 cytokines and eotaxin expression, and blocked Th2 cell differentiation without affecting Th1 cell differentiation. GS143 may be a candidate for the treatment of airway inflammation in asthma [92] (Fig. 2).

The serine/threonine phosphatase protein phosphatase-2A (PP2A) was abundant and its phosphorylation inhibited NF-κB signaling, reducing inflammatory responses. In allergic asthma, PP2A activity was reduced and correlated with the severity of the disease [93]. PP2A activators like 2-amino-4-(4-(heptyloxy) phenyl)-2-75 methylbutan-1-ol (AAL(S)) inhibit inflammation and AHR in allergic asthma, reducing eosinophil infiltration, mucus-secreting cells, Th2 cytokines, and serum IgE levels. In lungs, E3 ubiquitin ligases promote immune tolerance by degrading specific transcription factors, affecting Th2 cell activation [94]. Inhibitors of UPS (such as Bortezomib) reduced eosinophilic infiltration, airway inflammation, and Th2 cytokine production in allergic asthma, and inhibited AHR [95]. Combined therapy with AAL(S) and Bortezomib also reduced airway remodeling in chronic allergic asthma [96].

By enhancing MID-1 and limiting PP2A activity, TNF-associated apoptosis-inducing ligand (TRAIL) played a regulatory role in acute allergic airway inflammation by affecting the dephosphorylation of downstream pro-inflammatory signaling molecules. TRAIL^−/−^ mice showed lower levels of IL-4, IL-5, IL-10, and IL-13, and pro-inflammatory chemokines. Targeting TRAIL and its downstream pro-inflammatory signaling pathways, including PP2A, might help reduce airway remodeling features observed in chronic allergic airway inflammation [97].

Allergic asthma involves airway remodeling that is connected to the enhanced activity of the TGF-β signaling pathway. SMAD family member 7 (Smad7), Sloan-kettering institute (Ski) and Ski novel proteins (SnoN) were negative regulators of TGF-β signaling pathway [98], [99][98], [99] [[98], [99]]. Through the ubiquitin-dependent breakdown of Smad7, Ski, and SnoN, Arkadia, an E3 ubiquitin ligase, amplified TGF-β signaling, which promoted airway remodeling in allergic rats [100].

## 6 Discussion and conclusion

The regulation of allergic asthma by ubiquitination is a complex and multifaceted process that significantly impacts immune responses. In various biological processes, including immune functions, ubiquitination is vital for modulating signal transduction. Moreover, ubiquitination affects the growth, activation, and differentiation of T cells, thus ensuring effective adaptive immune responses to pathogens and tolerance to self-tissues. Dysregulated ubiquitination is linked to immune system disorders, including autoimmune and inflammatory diseases, highlighting its role in the pathogenesis of allergic asthma.

At present, proteasome inhibitors have been proven to be effective novel drugs for the treatment of multiple myeloma and mantle cell lymphoma [101], [102][101], [102] [[101], [102]]. Agents such as Bortezomib, Carfilzomib, and Ixazomib have been approved by the U.S. Food and Drug Administration (FDA)[103], [104], [105][103], [104], [105] [[103], [104], [105]] [[103], [104], [105]]. By modulating the UPS, it is hoped to alleviate the characteristic inflammatory responses and immune imbalances of asthma. However, the relationship between UPS and asthma still requires extensive investigation.

Firstly, the precise and safe targeting of UPS components remains a major challenge. In animal studies, GS143 demonstrated anti-asthmatic activity: intranasal administration significantly suppressed allergen-induced airway inflammation, eosinophil and lymphocyte infiltration, production of Th2 cytokines and chemokines, and reduced mucus secretion. However, its toxicity and side effects have not been investigated [92]. Bortezomib is a dipeptidyl boronic-acid small-molecule drug and the first globally approved inhibitor of the 26S proteasome. It reversibly binds the chymotrypsin-like active site of the proteasome, blocks the ubiquitin-proteasome pathway, and thereby prevents degradation of multiple short-lived and regulatory proteins [106]. Co-administration with AAL(S) can attenuate airway remodeling in chronic allergic asthma [96]. Similarly, its clinical applicability has yet to be thoroughly investigated. Currently, there is a lack of systematic studies comparing the efficacy, mechanisms, and side effects of these small-molecule inhibitors with those of glucocorticoids in allergic asthma, and this issue urgently demands investigation. Because the UPS regulates a vast number of proteins simultaneously, broad-spectrum inhibition may lead to off-target effects and systemic toxicity, limiting clinical application in asthma therapy [107].

Secondly, most research has focused on Th cells, especially Th2 and Th9 subsets, but there is limited understanding of how ubiquitination modulates other immune cells involved in allergic airway inflammation — such as B cells, innate lymphoid cells (ILCs), mast cells, and airway epithelial cells (even though we have listed some) [68]. These cell types may also contribute significantly to disease severity and airway remodeling but remain underexplored in the context of UPS regulation.

Thirdly, there is a critical need for more translational and clinical studies that bridge the gap between basic mechanistic insights and patient outcomes. So far, most evidence is based on in *vitro* experiments or murine models, while clinical trials investigating the effects of UPS modulators or E3 ligase inhibitors in asthma patients are scarce. Such studies are necessary to evaluate safety, dosing, efficacy, and potential biomarkers for patient stratification [107].

In summary, ubiquitination emerges as a key regulatory mechanism in allergic asthma, affecting immune responses through the modulation of various cellular and molecular pathways. Targeting these ubiquitination events could offer new avenues for therapeutic intervention in allergic asthma, emphasizing the need for further research into the complex interplay between ubiquitination and immune responses in allergic asthma.

## CRediT authorship contribution statement

**Zheng Jin:** Writing – original draft, Funding acquisition, Data curation. **Zhenhua Zhu:** Validation, Software, Investigation, Funding acquisition. **Xiaopeng Jing:** Visualization, Software, Investigation. **Ji Zeng:** Writing – review & editing, Supervision, Conceptualization. **Dongmei Yan:** Writing – review & editing, Validation, Supervision, Conceptualization.

## Declaration of competing interest

The authors declare that they have no conflict of interest.

All funding was used to advance the research, and there are no potential conflicts of interest that could affect the objectivity of the study.

## Data Availability

No data was used for the research described in the article.
